# Medicinal Plant Diversity and Inter-Cultural Interactions between Indigenous Guarani, *Criollos* and Polish Migrants in the Subtropics of Argentina

**DOI:** 10.1371/journal.pone.0169373

**Published:** 2017-01-11

**Authors:** Monika Kujawska, Norma I. Hilgert, Héctor A. Keller, Guillermo Gil

**Affiliations:** 1 Institute of Ethnology and Cultural Anthropology, University of Lodz, Lodz, Poland; 2 Instituto de Biología Subtropical, Universidad Nacional de Misiones—Consejo Nacional de Investigaciones Científicas y Tecnológicas, Centro de Investigaciones del Bosque Atlántico, Iguazú, Misiones, Argentina; 3 Facultad de Ciencias Forestales, Universidad Nacional de Misiones, Eldorado, Misiones, Argentina; 4 Instituto de Botánica del Nordeste, Consejo Nacional de Investigaciones Científicas y Tecnológicas, Corrientes, Corrientes, Argentina; 5 Centro de Investigaciones Ecológicas Subtropicales, Delegación Regional Nordeste, Administración de Parques Nacionales, Iguazú, Misiones, Argentina; Missouri Botanical Garden, UNITED STATES

## Abstract

Numerous studies highlight the importance of phytotherapy for indigenous and non-indigenous people in different parts of the world. In this work we analyze the richness (number of species), diversity (plant identity and the number of illnesses for which it is used) and similarity of plant species and illnesses treated with them, in order to contribute new data and insight into the importance of plant medicines to the local medical systems of people living in Misiones province, in the subtropics of Argentina. Three sympatric groups were compared: Guarani Indians, *Criollos* (mestizos) and Polish migrants. Quantitative scrutiny was focused on both primary and secondary sources. The similarity and diversity of medicinal plants and uses between groups was calculated by applying the Sørensen quantitative coefficient and the Shannon-Wiener index, respectively. In order to identify the characteristic plant species used by each group, the Cultural Importance and Prevalence Value (CIPV) was calculated based on the species Indicator Value (IndVal), which combines a species relative abundance with its relative frequency of occurrence in the various groups, and modified according to the type of the analyzed data. The important finding is a great variation in the number of species used by the study groups. Altogether, 509 botanical species were registered: Guarani (397), *Criollos* (243) and Polish migrants (137). For all groups, the use of native medicinal plants prevailed. The Guarani appear to be the local experts in use of medicinal plants. There is the significant difference in the number of treated illnesses by each taxon among three groups. *Criollos* and Polish migrants exhibit the greatest similarity in illnesses treated with medicinal plants. These groups share a corpus of knowledge related to illness nosology, and have a symptomatic approach to illness treatment. The Guarani have an etiological approach to illness diagnosis and healing, which may be viewed as a barrier to the exchange of knowledge about home medicine with other ethnic groups of Misiones.

## Introduction

Numerous studies within the fields of medical ethnobotany and ethnopharmacology highlight the importance of phytotherapy for indigenous and non-indigenous people in different parts of the world [1–4]. Morris [5] even argues that herbalism happens to be the most widespread and the most ancient form of therapy. Lambert and colleagues estimated that more than two billion people (approximately 34% of the world’s population at the end of the 20^th^ century) relied principally on medicinal plants for the treatment of illness [6]. Nevertheless, there is a lack of current exact estimations concerning the use of medicinal plant resources by local people across the globe [7]. Up to date quantitative estimations about the plants used in home and folk therapies are needed, especially those supported by a complex analysis of variables influencing the importance and persistence of plant medicines in local communities [8–11].

Measuring medicinal plant knowledge can give an insight into the cultural importance of plant resources, i.e. which species are recognized as effective, appreciated and reported with major frequency. Measuring this knowledge may also provide information about the proportions of agreement (consensus) and variation in medicinal plant use by groups within the same region, as well as distant but culturally similar groups [12–13]. Consensus analysis helps us to establish widely shared items within the semantic domain of a study and discover the accuracy of agreement of informants within this domain [13–14]. On the other hand, variation in knowledge between groups informs us about the particularities and distinctiveness of a given group [8]. Different methods have been developed by researchers and used to measure cultural importance, consensus and similarity [12]. Indices of cultural importance usually rely on the number of citations understood as one case of use of a specific species by a total number of informants within one cultural group, or informant-use report, i.e. a species used by an informant in a given use category or study domain [15–16]. Consensus indices, such as informant agreement ratio [17] and cultural agreement ratio [15] also require informant use-reports or number of citations. Quantitative analysis of consensus and similarity of species use between cultures can be also addressed by using diversity indices such as Shannon [18] and Sørensen [19]. Both these indices are apt for comparison among groups in different areas, but they are sensitive to variation in population size [12, 20].

What is the purpose of these attempts to develop adequate and precise techniques for the comparison of medicinal plant knowledge? The principal information contributed by comparative studies is the understanding of the reasons for which the groups who remain in a particular intercultural region maintain their own ways of curing and using certain species, which may be not appreciated by other ethnic groups, or may even be ignored by them. Comparative studies also help to establish which species are interchanged and for what reason [8, 12].

The comparative study of medicinal plant knowledge among sympatric ethnic and/or local groups may be divided into two types. The first type focuses on traditional groups with comparatively long residence in the region (*inter alia*: Heinrich et al. [8], Hilgert and Gil [21–22], Leonti et al. [23], Boer et al. [12]). The second type concentrates on the comparison of medicinal plant use by ethnic groups with different times of residence in a given region, such as the case of the native Italians and the historical Albanian community in southern Italy studied by Pieroni and Quave [24], and groups with different trajectories of migration process [25].

Diversity indices have been chosen in our analysis as we did not have full information about the number of citations per species use for all the sources we relied on in this comparison. Therefore all the informant data was pooled per ethnic group. The same procedure was adopted by Boer et al. [12], who studied the similarity in medicinal plant knowledge between three sympatric ethnic groups from Laos: the Brou, Saek and Kry.

In our case, three sympatric groups with different times of residence in the Misiones province, Argentina, and with partially different cultural backgrounds were chosen for comparison: 1) Guarani indigenous people belonging to the Tupi-Guarani linguistic family, with a long history of shifting cultivation and horticulture, 2) the mestizo people (called *Criollos* in Argentina), with their own identity and culture formed between the 17^th^ and 19^th^ centuries. These people have been penetrating Misiones from Brazil, Paraguay and the south of Argentina since the 19^th^ century; and 3) European peasants and farmers (depending on their status within the capitalist system in which they had been embedded in their country of origin), who arrived in Misiones en masse between the 1890s and 1940s. In this particular analysis, European migrants are represented by Polish settlers and their descendants, whose folk pharmacopoeia has been thoroughly studied [26]. It is to expect that people living in intimate contact with their surroundings for many generations gain in-depth knowledge of ecosystems in which they are inserted, and their management and utility, in this case about possible uses of medicinal plant species [12]. In this context groups that had shared the same geographical area and natural resources for a relatively long time, and were exposed to the same hazards, such as diseases, infections, and accidents for many generations would have evolved in the same way their empirical acquisition of medicinal plant knowledge [12]. In this study we hypothesize that: 1) the diversity of plants used in local medicine by Polish migrants, *Criollos* and Guarani is different; 2) *Criollos* and Guarani share more species with each other than with Polish migrants, due to their longer residence in the region, resulting in a greater familiarity with the local flora and a greater exchange of knowledge; 3) exotic species used by the three cultural groups are similar and are applied in a similar way, because they form part of a cosmopolitan pharmacopeia. Since cultural diversity in one region does not necessarily imply an increase in intercultural interactions [27], a general aim of this contribution is to analyze whether cultural diversity is reflected in the folk phytotherapy of these three groups from Misiones in Argentina. For this purpose, we analyze the diversity of medicinal plant uses by Guarani Indians, *Criollos* and Polish migrants in the Atlantic Forest of Misiones. Considering the three different cultural groups, our respective objectives were therefore: 1) to compare medicinal species used; 2) to compare exotic species used; 3) to compare the illnesses treated with plants; 4) to identify characteristic and important medicinal plants for each study group; 5) to identify and compare mostly shared species, and estimate the spectrum of illnesses treated with them; 6) to identify and compare illnesses according to their similarity, and estimate the number of species used in their treatment.

## Materials and Methods

### Study area and ethnographic setting

Misiones is a highly bio-diverse region with ecological habitat types characteristic of the subtropics. It is home to 3,000 vascular plant species. It forms part of a greater ecoregion known as the Atlantic Forest of the Upper Parana (*la Selva paranaense*) [28]. At the end of the 19th century this ecoregion extended over 1.2 million km^2^ from the Paraguay River to the Atlantic Ocean, covering eastern Paraguay, southern Brazil and the province of Misiones in Argentina. During the 20th century, the expansion of agriculture and animal husbandry, as well as deforestation, reduced it to 7.8% of its former size [29]. Misiones has preserved the largest continuous corridor of the Atlantic Forest in the Upper Parana: 57.5% of the original area [30]. The forest region covers 80% of Misiones, extending through its central and northern parts.

The study region from 1609 to 1767 was part of the ‘theocratic empire’ of the Jesuit missions, which gave their name to the modern province [31–32]. The missions were self-sustaining political, religious and economic organisms engaged in the evangelization and acculturation of the indigenous Guarani people [33]. With the expulsion of the Jesuits in 1767, the Guarani continued shifting agriculture and horticulture [34]. In the 19^th^ century mestizo people were visiting Misiones for selective extraction of timber and collection of wild yerba mate. In 1897, the first European immigrants arrived in the south of Misiones. They were of Polish and Ukrainian origin [35]. The process of populating the province with European peasant families continued until the 1940s, varying in character but basically relying on an ethnic pattern of settlement (e.g. Swiss colonies, German colonies, etc.) [36]. Since the 19^th^ century, *Criollos* have been coming to Misiones from the south of Argentina (Corrientes) and from the neighboring Brazil and Paraguay; the flow of Paraguayans has increased dramatically in recent years [37]. Guarani people have been resident the longest in Misiones, nevertheless they are the least numerous ethnic group, consisting of approximately 3,500 persons [38]. Guarani dedicate themselves to subsistence horticulture, craftwork and seasonal work on the farms of European descendants and *Criollo* people.

### Illness concepts

For the Guarani, etiologies have a predominantly personalistic character (*sensu* Foster [39]). An illness may be the outcome of the activity of witches, ghosts, or owners of species and elements of nature, all considered to be beings with vast, relevant attributes in the social reproduction of the Guarani communities [40]. The condition for the socializing of these pathogens places them in a ‘litigation setting’, preventing even the mildest health problems from being underestimated as simply symptomatic (or naturalistic, *sensu* Foster [39]). The healing process, therefore, implies the restoration of a social order, in which diagnosis and treatment are both indispensable and interrelated. Hence medicinal plants used in health treatment can be conceived more as conveyors linking a person (a patient) to ancestors, who have been transformed into archetypes or owners of the used plants [41]. *Criollos* and Polish migrants share the corpus of knowledge related to illness nosology. Both groups have a symptomatic approach to illness treatment. Hence they are not guided by etiologies to treat their illnesses, but rather focus on symptoms [42].

### Ethics statement

The authors followed all the requirements and regulations needed for conducting ethnobiological fieldwork in Argentina. Oral informed consent was obtained from each participant involved in this study. Information provided by all participants was kept confidential and was anonymously tracked using an identification number. The required permissions for collection of references materials were obtained from the Provincial Ministry of Ecology in Posadas, Misiones. This permission has been renewed every year with the appropriate authorities. No ethical approval was required for this study in Argentina, as no participants were subjected to any other treatment than voluntary interviews. The research was carried out following the code of ethics of the American Anthropological Association and the International Society of Ethnobiology Code of Ethics (http://www.ethnobiology.net/what-we-do/core-programs/ise-ethics-program/code-of-ethics/, accessed 08.07.2015).

### Data collection

In this study we employed original data from our previous research, which has been partly published, and secondary sources, too. The information concerning the use of medicinal plants among *Criollos* and Polish migrants in Misiones has been already published, however the data about the phytotherapy of Guarani people still remain in the format of a doctoral thesis [38]. The information from secondary sources was obtained from the medical ethnobotanical literature concerning the northern part of Misiones, coinciding with our study area [43]. In this analysis we excluded the southern part, phytogeographically different from the rest of Misiones. This was also due to the heterogeneous character of data from the south of Misiones–we could not ascribe them to a particular ethnic group (they were collected in the city markets of Posadas, the capital of Misiones) [44].

Ethnographic material concerning Guarani people was gathered in 12 Guarani indigenous communities in central Misiones, in the departments of Libertador Gral. San Martín, San Pedro, Eldorado and Guaraní [38]. The author worked with a total of 83 Guarani women and men, ages ranging from 8 years old to senior persons of over 70 years old. The information was obtained through open interviews in Spanish and Guarani, surveys, records of testimonies and legends, participant observation, joint review of herbarium specimens and walks in the forest with mobile interlocutors (Table 1).

**Table 1:** The number of participants taken into account for data analysis related to each cultural group.

Study group	Nr of participants	Period of fieldwork	References
**The Guarani**	83	1997–2008	Keller [38]
**Criollos of Brazilian origin**	65	2005–2006	Keller and Romero [45]
**Criollos of Paraguayan origin**	14 + 23	2005–2010	Moreau [43], Kujawska et al. [46]
**Polish migrants and their descendants**	94	2007–2011	Kujawska and Hilgert [26]

**Fig 1:**
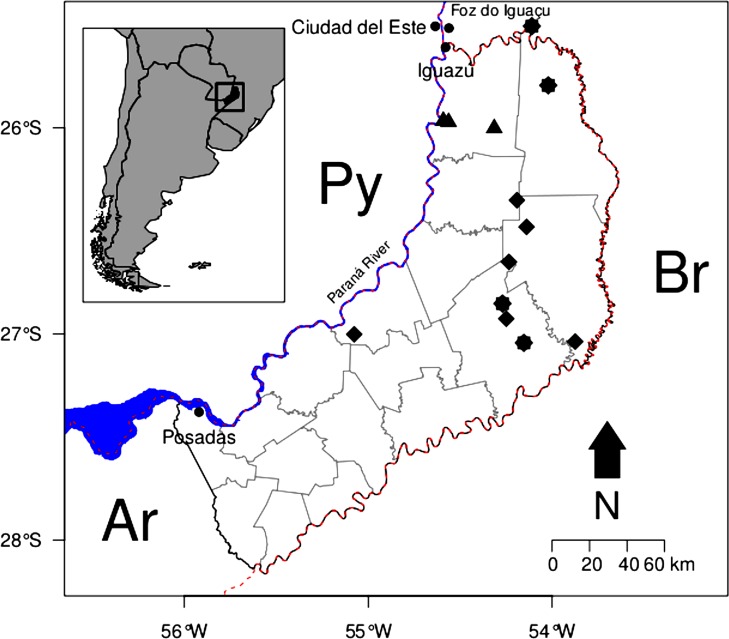
Study area: the localities in the Misiones province in Argentina where field research among Guarani Indians, *Criollos* (mestizos) and Polish migrants was conducted.

The data about use of medicinal plants by *Criollos* was obtained from three ethnobotanical studies [43, 45–46]. Keller and Romero [45] conducted their study among small farmers living near the Reserva de Biósfera Yabotí, in the central-eastern part of Misiones. The majority of interviewed farmers were of Brazilian origin. The interviews had a semi-structured character and were directed to 65 people. Moreau [43], Kujawska et al. [46] and Zamudio et al. [47] carried out their research, respectively, among small farmers from the department of General Manuel Belgrano–the extreme northern part of Misiones. The interviewed *Criollos* were predominantly of Paraguayan origin, dedicating themselves to subsistence farming and the cultivation of tobacco as a cash crop, with animal husbandry as a supplementary activity. Moreau worked with four key informants, who were subject to in-depth interviews, and 10 other interlocutors (4 women and 10 men in total). Meanwhile, Zamudio directed structured questionnaires to 23 *Criollos* people (9 women and 14 men, aged between 20 and 70) (Fig 1).

Ethnographic material concerning Polish migrants was collected by applying semi-structured, in-depth and free listing interviews to 94 study participants (62 women and 32 men, aged 28–92) between 2007 and 2011. The researched group consisted of Polish migrants and their descendants, mainly from the first generation born in Misiones. Polish migrants arrived in northern Misiones and settled in the rural colonies of Wanda and Lanusse between 1936 and 1938. They originated from rural areas in eastern Poland, and in Misiones also dedicated themselves to land cultivation complemented with animal husbandry [26, 46] (Fig 1).

All the analyzed botanical species were supported with voucher specimens. The specimens were identified by the authors and deposited in the herbarium CTES of the Instituto de Botánica del Nordeste (IBONE) in Corrientes. Plant names and authors were verified with the Plant List database (http://www.theplantlist.org/, accessed 08.07.2015).

### Data analysis

With the list of medicinal plants and associated illnesses treated by the three groups we estimated the number of species (the species richness, in ecological terms [48]) from the local and introduced flora, which are used in health treatment by inhabitants of central and northern Misiones.

The analysis of plant and illness richness, similarity and diversity [48] was performed twofold. First we concentrated on all plant species and all illnesses reported by each study group (objectives 1–4). Then, in the second phase, we confined our analysis to the botanical species and illnesses that were shared by the three cultural groups (objectives 5–6).

In order to compare all the species used by each study group, and exotic plants in particular (objective 1 and 2), three different approximations were made: first, we counted the number (richness) of species used by each group. Second, we calculated the similarity in the number of illnesses treated by a given plant species for each pair among the study groups by applying the Sørensen quantitative coefficient [48]. A maximum value (100%) means that both groups use the same plants for the same number of illnesses, and a minimum (0%) means that the compared pairs do not share any medicinal plant species. The third measure was the diversity of shared species, calculated with the Shannon-Wiener index (H values) [48]. Only species shared by all study groups were taken into account, in order to compare the diversity of uses of the same species in different cultural groups.

In order to assess whether the differences in diversity found were statistically significant, we compared H values by applying modified t test, based on the normal distribution of values that h statistic takes (t values with df–degrees of freedom) [48–49]. Species diversity is a parameter widely used in community ecology [48]. It takes into account species richness (number of species) and their relative abundance, to create a composite value. This diversity index is also used in ethnobiological studies, where it is calculated for used species (use richness), while relative abundance is replaced by frequency of citations [8]. In our analysis we did not take frequency of citation into account, due to the lack of the relevant information from some of our sources. Therefore, we propose an alternative comparative analysis of use diversity, which takes into account the number of illnesses treated by each species, instead of frequency of citation. The proposed approach can be further applied to the analysis of different type of data, especially historical ethnomedical sources, which usually lack a measure of frequency of citation by informants. We tested the relationship between illnesses and cultural groups by applying a Chi-square test [49], taking the number of plant species used as a variable (objective 3).

In order to identify the characteristic plant species used by each group, the Cultural Importance and Prevalence Value (CIPV) was calculated (objective 4). We can recall only one other ethnobotanical study in which this index has been applied [50]. It was based on the species Indicator Value (IndVal) proposed by Dufrene and Legendre [51], and modified according to the type of data analyzed. For each study group, the index measures constancy of use of each species, taking into account the number of health conditions treated within each body system (here understood as a category of use), as well as the degree of exclusivity with which the species is used by each group. A high value of CIPV is achieved when a given species has many uses for the possibly greatest number of body systems, and at the same time is used exclusively, or nearly exclusively, by a particular cultural group. A low value is observed when a species has few or no applications and/or a species is not exclusive to a given cultural group, but is also frequently used by at least one other group. The final formula is as follows: CIPV = Aij x Bij x 100, where i = used species, j = a cultural group, A = a measure of specificity of uses, B = a measure of fidelity observed in these uses. Final multiplication by 100 produces percentages. The first component of the formula is as follows: Aij = Ndiseij/Ndisei (dise stands for disease), where Ndiseij = the average number of treated illnesses for a body system with species i by cultural group j, Ndisei = the sum of Ndiseij of all cultural groups. The second component of the formula is as follows: Bij = Nsystij/Nsystj, where Nsystij = number of body systems treated with species i by cultural group j, Nsystj = total number of body systems treated by cultural group j. In order to determine whether the obtained CIPV values were significantly different from those that could be obtained by random distribution of body systems amongst different groups (the observed data and those expected by chance), we used an average of 1,000 permutations (z statistic). Only species with CIVP p <0.05 were considered.

From all the plant species mentioned by the three study groups, we selected those that were the most extensively shared by the Guarani, *Criollos* and Polish migrants, based on the number of illnesses treated (objective 5). In other words, only plant species that were shared by three study groups and that were used in the treatment of the greatest number of illnesses were chosen for this comparison. We followed the same analysis scheme in order to establish the illnesses which were the most highly shared by the study groups (objective 6). In these cases, we also used the Sørensen quantitative coefficient of similarity and the Shannon-Wiener diversity index, and for the latter the observed numbers were compared with the modified *t* test [48].

## Results

### Medicinal plant richness

In total, we registered 509 plant species used in home phytotherapy by three cultural groups from the study region (objective 1 and 2). However, the number of species used as medicines varies between groups. The Guarani use 397 (337 native, 60 exotic) species in their home therapies, while *Criollos* reported 243 (157 native, 86 exotic) plants as medicinally useful. Polish migrants and their descendants use 137 (75 native, 62 exotic) botanical taxa. For all study groups, the use of native medicinal plants prevails, however the exotic species are applied in different proportions by each group: for the Guarani it is only a fraction– 15%, while for Polish migrants exotic species account for 45% of all used species.

**Fig 2:**
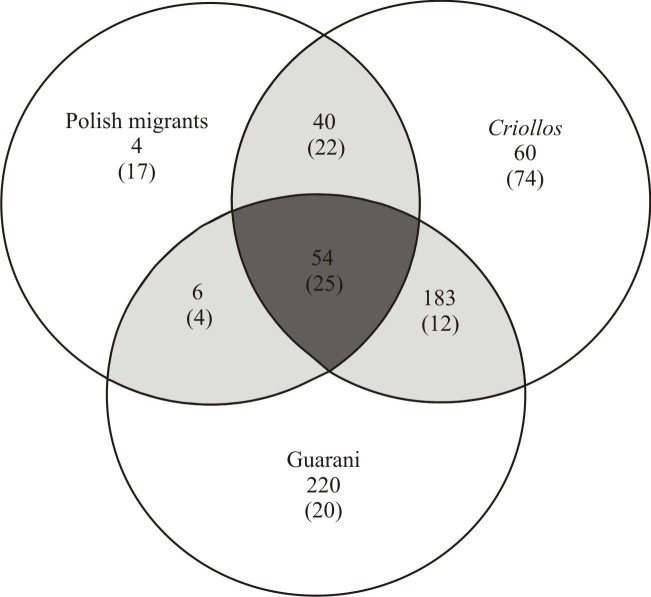
Exclusive and shared medicinal plant species registered among the Guarani, *Criollos* and Polish migrants. ### = native species, (###) = introduced species.

The list of medicinal plants used by the Guarani is 39% and 66% longer than that of *Criollos* and Polish migrants, respectively. *Criollos* mentioned 44% more species used in their therapies than Polish migrants did (Fig 2).

### Similarity and diversity of medicinally useful plants

**Fig 3:**
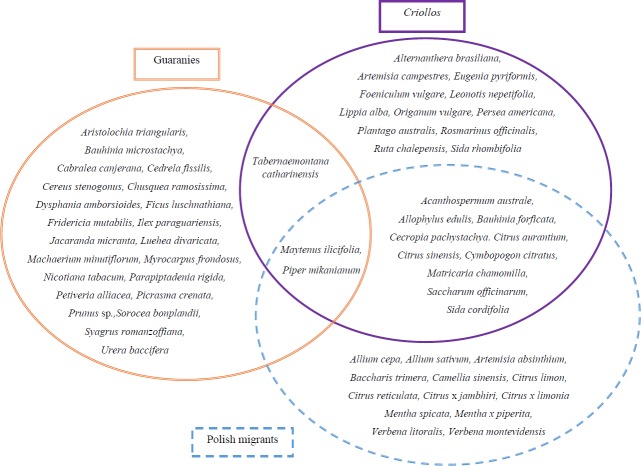
The exclusive and shared most versatile plant species used in the treatment of the largest spectrum of illnesses by the Guarani, *Criollos* and Polish migrants.

In order to estimate species similarity and diversity (objective 1), we considered the number of medicinal uses assigned to each botanical taxon. By analyzing species similarity between the study groups, we observed that *Criollos* and Polish migrants are more similar to each other (45%), followed by *Criollos* and Guarani (32%), and finally Guarani and Polish migrants (19%). Species diversity is the highest for the Guarani group (H = 5.53), followed by *Criollos* (H = 5.22), and the lowest for Polish migrants (H = 4.54), with significant differences (p<0.05) between each of the pairs (Guarani-*Criollos*: t = 163, df = 8934; Guarani-Polish: t = 93, df = 632; Polish-*Criollos*: t = 91, df = 689) (Fig 3).

While comparing exotic species exclusively, (objective 2), Polish migrants and *Criollos* exhibit the greater similarity (52%), and the similarity between two other pairs: the Guarani and *Criollos* (34%) and the Guarani and Polish migrants (31%) are maintained at nearly the same level. The diversity is the highest for *Criollos* (H = 2.05), with lower and more similar levels for Guarani (H = 1.81) and Poles (H = 1.73), while significant differences (p<0.05) between each of these pairs are maintained (Guarani-*Criollos*: t = 63, df = 587; Guarani-Polish: t = 45, df = 734; Polish-*Criollos*: t = 52, df = 442).

### Similarity of illnesses

In order to estimate the illness similarity between study groups, we considered the number of medicinal plant uses ascribed to each health condition (objective 3). The similarity between *Criollos* and Polish migrants was relatively high (79%), in comparison with *Criollos* and Guarani (22%), and Guarani and Polish migrants (24%). The number of species used in the treatment of illnesses by each study group was significantly different (p<0.05) (Guarani-*Criollos*: Chi2 = 1215, df = 219; Guarani-Polish: Chi2 = 1035, df = 205; Polish-*Criollos*: Chi2 = 20184, df = 161) (Table 2).

**Table 2:** A ranking of 25 illnesses treated with the largest number of plant species by each cultural group.

Illnesses treated	Guarani	*Criollos*	Polish migrants
**Fever**	74*	16	5
**Cough**	73	44	19
**Diarrhea**	61	13	14
**Wounds**	50	32	16
**Toothache**	47	16	5
**Intestinal parasites**	43	15	9
**Digestive problems**	28	**53**	**24**
**Flu**	24	44	21
**Headache**	58	18	
**Kidney infection**	24	39	
**Body ache**	29		8
**Menstrual pain**	27		11
**Hypertension**		26	12
**Contaminated blood (blood cleanser)**		24	23
**Liver pain**		23	12
**Gastric hyperacidity and or ulcers**		18	5
**Common cold**		14	17
**Sore throat**		14	9
**Agitation, nervous tension**		13	12
**Heart illnesses**	37		
**Asthma**	32		
**Scabies**	31		
**Measles**	29		
**Dizziness**	28		
**Foot swelling**	28		
**Epilepsy**	26		
**Eye infection**	25		
**Shooting pains**	24		
**Physical weakness**	23		
**Back pains**	22		
**Pimples on scalp**	22		
**Snakebite**	22		
**Infections**		18	
**Diabetes**		16	
**Muscle aches**		16	
**Urinary tract infection**		15	
**Circulatory problems**		14	
**Intestinal infections**		13	
**Angina**		12	
**Tonic**		12	
**Stomach ache**			16
**Bronchitis**			15
**Catarrh**			12
**Cold sore**			8
**Prophylactics**			8
**Empacho**			7
**Flatulence**			7
**Insomnia**			6

### Culturally characteristic medicinal species

By identifying characteristic and important plant species for each cultural group (objective 4), 82 species achieved Cultural Importance and Prevalence (CIP) value for Guarani people (with values between 55 and 18) the most important being: *Syagrus romanzoffiana* (55), *Cabralea canjerana* (52), *Petiveria alliacea* (51), *Prunus* sp. (48), *Aristolochia triangularis* (48), *Nicotiana tabacum* (42) and *Hennecartia omphalandra* (41). Eight species with the highest CIP values resulted to be characteristic and prominent for *Criollos*: *Plantago australis* (27), *Persea americana* (24), *Citrus aurantifolia* (23), *Artemisia campestris* (22), *Rosmarinus officinalis* (21), *Ceiba speciosa* (18), *Rubus sellowii* (18) and *Solanum sisymbriifolium* (18). Three species were identified as characteristic and the most important for Polish migrants: *Mentha* x *piperita* (19), *Camellia sinensis* (16) and *Mentha spicata* (16).

### Mostly shared species

**Fig 4:**
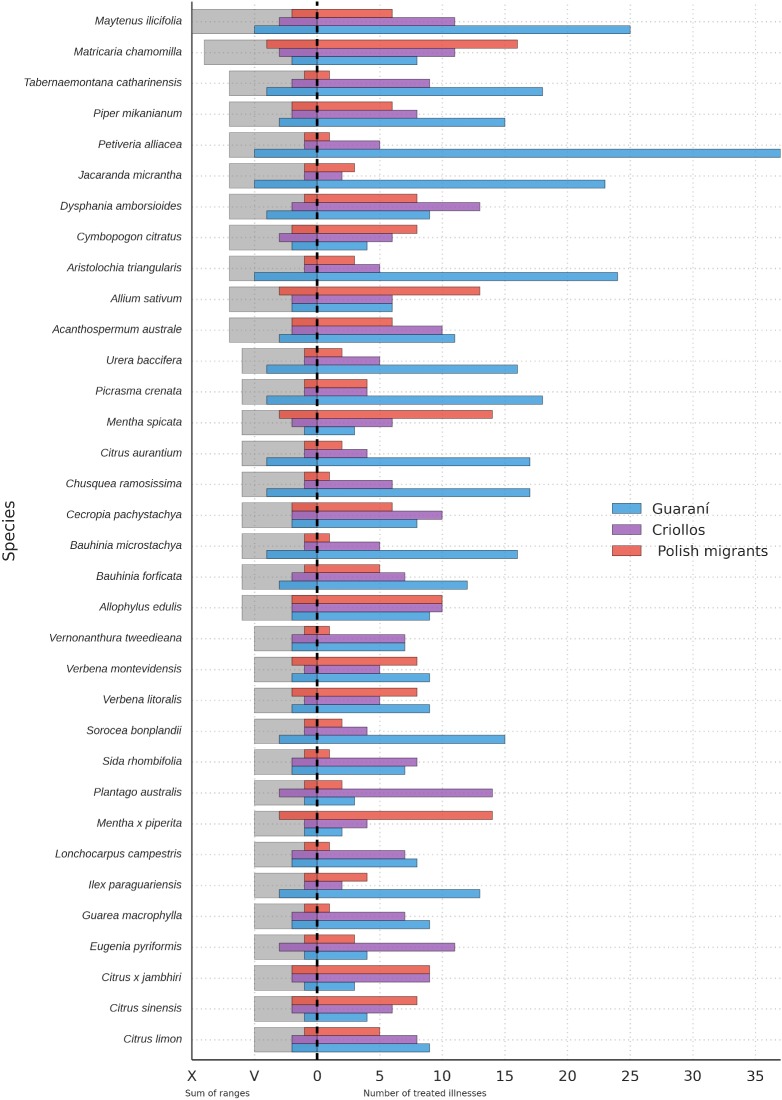
Botanical species shared by the three cultural groups, which have the most diverse (versatile) uses. These species were categorized according to number of illnesses treated by the following scale: 1–5 illnesses = I; 6–10 illnesses = II; 11–15 illnesses = III; 16–20 illnesses = IV; 20 or above illnesses = V. These categories were then summed up for each species and only species that achieved value equal to five or higher were selected.

The analysis of the species mostly shared by the three groups and the spectrum of illnesses treated with them showed that *Maytenus ilicifolia* and *Matricaria chamomilla* treat the largest number of illnesses, considering the three groups together. The native *Petiveria alliacea* is the most versatile species for the Guarani (used in the treatment of 37 different illnesses); the native Polish plant–*Matricaria chamomilla*, exotic in Misiones, is used as the most versatile medicinal plant by Polish migrants (16 different illnesses), and *Plantago australis* is the most versatile medicinal resource for *Criollos* (14 illnesses). By comparing the mostly shared species (objective 5), we observed that *Criollos* and Polish migrants were the most similar to each other (66%). The second most similar pair were *Criollos* and the Guarani (61%), and finally the Guarani and Polish migrants (45%). The diversity of health conditions treated with the pool of plant species reaches a similar value between groups (H = 3.22–3.44), but significant differences (p<0.05) remained between each of these pairs (Guarani-*Criollos*: t = 86, df = 1204; Guarani-Polish: t = 53, df = 329; Polish-*Criollos*: t = 55, df = 287) (Fig 4).

### Mostly shared illnesses

**Fig 5:**
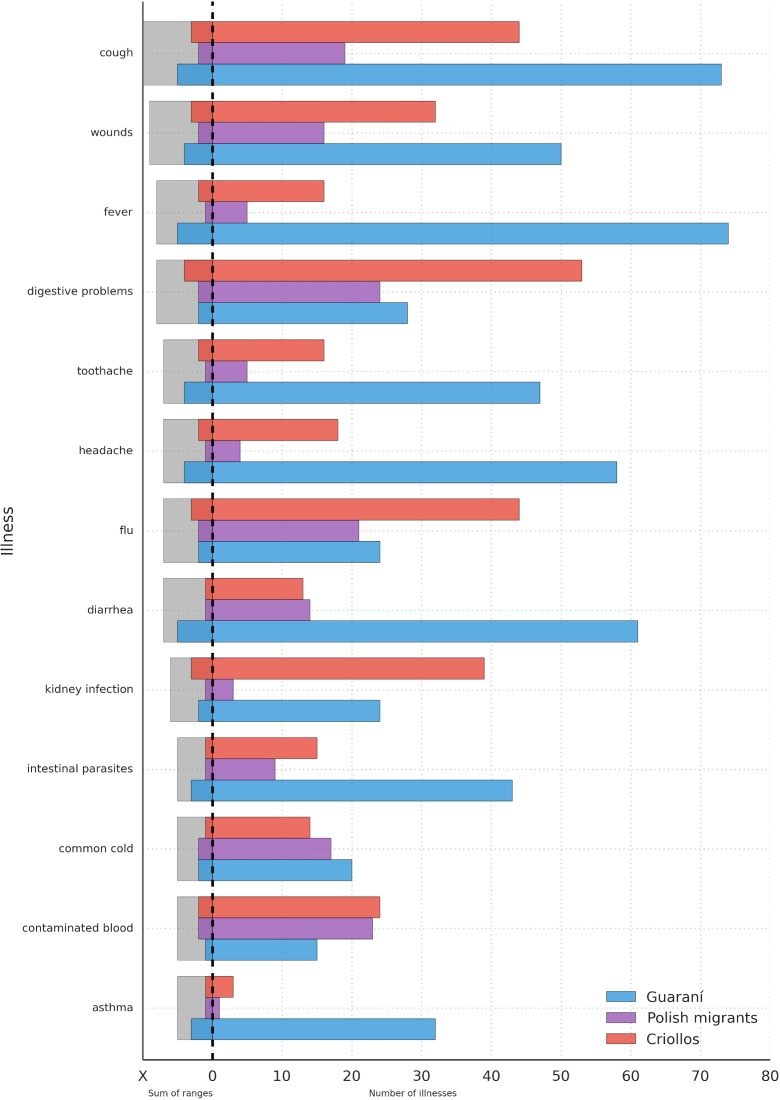
Illnesses shared by the Guarani, *Criollos* and Polish migrants and treated with the largest number of species. The chosen illnesses were categorized according to the following scale: 1–15 species = I, 16–30 species = II, 31–45 species = III, 46–60 species = IV, 61–75 species = V. These categories were then summed up for each illness, and only those illnesses that achieved value equal to five or higher were selected.

In relation to the illnesses shared by the three groups and the number of species used in their treatment, we observed that coughs and wounds are the most shared health problems. The Guarani deal with the same complaints using the greatest number of species, followed by *Criollos*, who use the largest number of medicinal plants from all groups in the treatment of digestive problems, flu and kidney infections. Using this parameter (objective 6), *Criollos* and Polish migrants are the most similar to each other (64%), followed by the Guarani and *Criollos* (59%), while the Guarani and Polish migrants display the lowest similarity (43%). The diversity of species used per illness reaches similar values between groups (H = 2.33–2.45), a significant difference (p<0.05) remain between each of the compared pairs (Guarani-*Criollos*: t = 68, df = 6383; Guarani-Polish: t = 48, df = 224; Polish-*Criollos*: t = 43, df = 332) (Fig 5).

## Discussion

### Similarities in use of medicinal plants among sympatric groups

This paper represents a new insight into the processes of interaction in a multicultural region of Misiones. To our knowledge, this is the first analysis of this type performed in the Southern cone of South America [8, 12, 24, 52]. The results partially confirmed our hypothesis. Indeed, we found a large variation in the number of species used by the study groups, as well as in the number of uses assigned to them. The greatest diversity of medicinal plants used was found among the Guarani, followed by *Criollos* and Polish migrants. However, *Criollos* and Polish migrants were more similar in their use of medicinal plants than any other compared pair. In the same fashion, the illness similarity between *Criollos* and Polish migrants was relatively high. Finally, with respect to the use of exotic species, we did not see any major similarity between the three cultural groups. The performed comparison is only valid when a similar approach is adopted during the research among the study groups. In the ecological context, the measures used (Shannon-Wiener, Sørensen, CIP) would require similar sampling. This prerequisite is valid for our comparison, as we are perfectly aware of the research techniques, population samples, places and periods of fieldwork, being ourselves the authors of the source papers used in this analysis.

Other studies dedicated to the comparison of medicinal plant knowledge among sympatric groups report results similar to ours. Boer and colleagues [12] stated that three indigenous groups living in the same region of the Annamite Mountains near the Vietnamese border in the Lao PDR maintained a low similarity in plant use, considering that the groups shared the same ecological area, had the same dependence on medicinal plants, and preserved a similar mode of subsistence. A possible explanation for this fact is that a considerable portion of remedies used within each ethnic group are important on cultural (symbolic) grounds, but prove ineffective pharmacologically. Medicinal plants, unlike domesticated food plants, are likely to offer an effective cure for a limited number of illnesses, and additionally may be toxic. Therefore, when borrowings are made from one culture to another in the use of medicinal plants, these resources are normally tested for their effectiveness by the borrowing group, rather than being embraced automatically due to their potential high cultural or symbolic value in the culture from which they are borrowed. According to this paradigm, the importance of medicinal species may evolve differently in a culture which has been exposed to prolonged contact with a given resource, than among a local/ethnic /migrant group which is in the process of adopting a given species and is placing it within its own ethnomedical frame. Collins et al. [52] reported an even lower similarity in plant use between two East Timor ethnic groups: the Laklei and Idate, who live in a short distance from each other (10–20 km) and are surrounded by virtually identical flora. These groups used 44 and 53 medicinal plants respectively, and shared only 11 of these taxa. The shared taxa are well known in the whole of South East Asia, and have known bioactive components. A possible explanation is the minimal cultural exchange of knowledge due to an ecological barrier–a mountain range between the two groups. The study by Leonti et al. [53] presents a similar scenario of low similarity between two groups, in this case living in the Olmec region, namely Lowland Mixe and Zoque-Popoluca. In this context, the study performed by Pieroni and Quave [24] among a historical Albanian migrant group and native Italians living in the same region of South Italy contrasts with our findings and those of other researchers. As the authors explain it, the relatively high similarity is possibly the outcome of an acculturation process the Albanian diaspora went through in Italy over the last five centuries. These results, however, do not stay in the line with our finding, in the sense, that *Criollos*, who have the relative long residence in the region, share more medicinal plant species with the relatively new group of Polish migrants who settled in the area 80 years ago than with the Guarani.

Our first finding is the great species richness used by the Guarani, who appear to be local experts in medicinal plants, especially native ones. In the case of Guarani people, their longest residence in the region translates into a thorough familiarity with the local flora and coincides with the most diverse use of medicinal plant species. This pattern, however, does not seem universal, as in the Great Chaco region, for example, indigenous people also have the longest residence, but their lists of medicinal plants are shorter than those of *Criollos* people from the same ecoregion. Researchers relate this phenomenon to the fact that Chaco indigenous people (Ayoreo, Maká, Pilagá) still visit shamans to treat illnesses, and shamanic curing does not normally include the use of medicinal plants, or at least not directly [54–57]. Therefore, it is not only the time spent in one particular region which matters, but also the place of medicinal plants in the overall ethnomedical system of the given group, as well as access to botanical resources. A high richness of plant and medicinal uses was also documented among other sedentary groups of the Tupi-Guarani linguistic family. In relation to this attribute found among the Amazonian Ka'apor, Balée [58] suggests that despite colonial impact, this group has been able to maintain its cosmovision and knowledge of phytotherapy. These findings contrast with the above mentioned reports from the Chaco region and the more generalist conjecture from the Amazonia region [59] that pre-colonial Amerindians used relatively few plant medicines to treat a limited number of illnesses due to their major reliance on shamanic healing. The diversity of phytotherapy of sedentary Tupi-Guarani seems to constitute an ethnic quality deeply rooted in their cosmological foundations. However, it is very likely that this quality has been nurtured and multiplied by a resilient response to the new pathological disorders established after colonization and contact with national society.

### Illness concepts and their relation to medicinal plant use

The second important finding of this study is the significant difference in species diversity in relation to the number of illnesses treated among the three groups. If we take into account the number of illnesses treated, we find that *Criollos* and Polish migrants share more medicinal species and exhibit a greater similarity in modes of using them. This is due to a shared corpus of knowledge related to illness nosology: both groups have a symptom-specific approach to illness treatment, which may facilitate knowledge exchange about illness treatment, including knowledge about medicinal plant use. Some authors suggest that the intercultural sharing may be explained by the pharmacological effectiveness of shared medicinal plants. Symbolic use is culture dependent and is less likely to be borrowed from one ethnic group to another [12].

It is expected that in the near future Guarani people will incorporate some of the illness concepts shared by *Criollos* and Polish migrants, which will make the flow of knowledge more equitable. This may happen for two reasons. Firstly, Guarani people visit health centers with increased frequency, both hospitals and first aid ambulatories, where they learn about chronic diseases such as hypertension, high levels of uric acid and high levels of cholesterol, diabetes and the like [60]. Our field observations indicate that both *Criollos* and Polish migrants actively search for medicinal plants (native and exotic) to alleviate these health problems. This is partly due to the fact that for both Polish settlers and *Criollos*, herbal medicine is the preferred form of treating illnesses, and they exhibit reluctance towards using pharmaceuticals for the treatment of chronic diseases [26, 42]. Hence both *Criollos* and Polish migrants may become an important source of knowledge about natural medicine to treat chronic diseases for the Guarani, especially in finding new applications for already known plant medicines (from the local flora), as well as from the “global stream”. However, in general terms, Guarani people preserve a different approach to illness diagnosis and healing, which consequently may be viewed as a barrier in the exchange of knowledge about home medicine with other ethnic groups of Misiones. This group’s phytomedicine is closely interconnected to the magical uses of plants, often making it difficult to make a clear demarcation between these two categories [61], and it is likely that many medicinal uses have derived from magical applications. For example, in order to treat depressive states, the Guarani ingest a decoction of *Chaptalia nutans*. In Guarani cosmology, this plant is guarded by an archetypal protective entity, which since its mythical metamorphosis, is dedicated to helping alleviate depressive states caused by marital problems. The normal procedure of transaction with this entity does not involve ingestion of the plant [41]. As evidenced by the results of this contribution, the Guarani’s way of addressing health problems hampers, but does not prevent, the exchange of plant resources and therapeutic procedures by Guarani individuals who establish cursory contact with their *Criollos* and Polish neighbours. However, the transfer of medicinal species used by *Criollos* into the cosmological corpus of the Guarani is not implemented by a simple inclusion of knowledge about their therapeutic actions. For example, *Criollos* cultivate a species called “corazón de San Francisco” (*Leonotis nepetifolia*) in their gardens, to which they ascribe various therapeutic properties [45]. The transplantation of this species into the *patios* of the Guarani is not merely an outcome of the recognition of its therapeutic virtues, but rather due to the capacity of the flowers of this species to attract hummingbirds. These birds are considered emissaries of the archetype of lightning, one of the principal deities of Guarani people [62]. Here, the cultural value of a medicinal plant is measured in relation to its capacity to connect with benefactor deities. For these reasons, the question of what makes a plant a culturally important resource can be answered by referring to the inherent scale/logic of relevance of each ethnic group. As we can observe, the study groups have different characteristic and important plant species. In a study similar to ours–comparing the use of medicinal plants in the four indigenous groups of Mexico, Heinrich and colleagues [8] stated that all culturally important species were aromatic and rich in essential oils. This plant characteristic may indicate a sensory perception of the environment and give some idea of the plant selection patterns used by Mexican Indians. However, in our study region we do not observe the same clear patterns for the selection of medicinal plants.

### Cultural responses to medicinal plant diversity in an intercultural context

Our findings reveal that the Guarani have numerous plant species to treat a particular health condition, e.g. they use, on a community level, 76 different species to treat fever, 61 different plants to treat diarrhea, 26 different species for epilepsy, and so forth. The Guarani also distinguish more than two hundred different illnesses and possess a considerable range of modes to treat them with herbal medicines [38]. The deductively formulated reasoning for this case may be that a low intra-cultural consensus and a multitude of medicinal species indicate the lack of an effective cure and a constant and evolving search for accurate remedies (see Boer et al. [12]). However, we propose an alternative explanation that the versatility of medicinal plant uses may be viewed as a cultural strategy. Moreover, the great number of medicinal plant species used to treat one particular illness is likely to be due to intra-cultural knowledge distribution, which is very individualistic among the Guarani from Misiones [38], rather than the use of hundreds of ineffective plant medicines.

Polish settlers managed to conserve some of the most important species from their native culture, especially some ubiquitous species, previously introduced by other Europeans to the region or brought from the country of origin [26, 63]. This phenomenon was confirmed by our analysis and reflected in the most characteristic and important species used by this group. It may be hypothesized that plant medicine incorporated by other ethnic groups from Polish migrants might be based only on pharmacological effectiveness, as this group did not present an attractive source of knowledge in this domain for either Guarani or *Criollos*. It is worthy of note, that the great majority of medicinal exotic species used by Polish migrants had previously been introduced by Spanish, Italian and German, who had longer migratory trajectories in Argentina. However, pharmacological assessment of the plants used by the three groups was beyond the scope of this research.

At first glance, it looks as if *Criollos* were intermediaries between the indigenous people and European migrants. Although possessing strong bridging capital [64], this group has developed knowledge and uses of medicinal plants *sui generi*. This can be observed in the rather low similarity between them and other study groups in the diversity of used species, and exotic ones in particular. *Crioll*os may be migrants in Misiones but they have a quite long history themselves and a relationship with the rural environment which goes back nearly 500 years in the Southern cone.

### Medicinal plant review in the american southern cone

This kind of ethnopharmacological review is here performed in Argentina for the first time. The only other known review of medicinal plant uses on a regional scale was conducted by Molares and Ladio [65], but it was confined to a single ethnic group–Mapuche people. The author described the use of 505 medicinal species registered in 16 publications from Argentina and Chile during the period 1955–2007. Viewed more generally, on an ecoregion scale, our study is the first of this type carried out in the Interior Atlantic Forest. However, the exhaustive study of Begossi et al. [2] performed in another ecoregion–the Brazilian part of the Atlantic Forest complex should be mentioned. This analysis was confined to one multi-ethnic group, the *Caiçaras* (descendants of native Tupinamba Indians and Portuguese settlers), equivalent of *Criollos* from our study region. The *Caiçaras* mentioned altogether 227 medicinally useful plants. Begossi and colleagues [2] underlined that the *Caiçaras* used a relatively wide range of medicinally useful plants for a relatively high number of illnesses, demonstrating a wide and deep knowledge of medicinal plant properties. This conclusion generally coincides with our findings too, with one difference. Brazilian researchers observed that *Caiçaras* predominantly used plants of foreign origin, which was not noted in our study. This prevalence of exotic medicinal plants was explained by their multi-ethnic character and strong European legacy, by little contact with Guarani Indians, and the advanced state of deforestation in that part of Brazil. Contrary to this trend, in central and northern Misiones European migrants who had settled in an isolated forested area, had to adapt to local conditions and incorporate local flora for treating illnesses. Therefore their cultural legacy, as well as the use of their native plants did not produce much effect on other ethnic groups of Misiones. It is probably a combination of the small impact of Polish migrants on the overall body of knowledge of medicinal plants in the region, the relatively good preservation of the Atlantic Forest throughout the 20^th^ century, and relatively intensive contact with Guarani people and *Criollos*, that led to the importance of native medicinal plant species for all the study groups in Misiones.

## Conclusions

Medical ethnobotanical studies nowadays are much more than the compilation of a list of medicinally useful plants. Several methods have already been proposed for the intercultural comparison of uses of medicinal plants. This paper’s methodology presents a special case, as some data are missing, such as frequency of citations. In this case all the informant data was pooled per ethnic group and diversity indices were applied for comparison analysis of three sympatric groups inhabiting one highly bio-diverse region.

With this paper we contribute to the up-to-date estimations of medicinal plants used by the indigenous and migrant groups living in South America. The 509 botanical species used medicinally presents a relatively high number, most of which are native to flora of the Southern cone. This particular finding counters the notion of the erosion of medicinal plant knowledge among indigenous and folk societies across the globe, widely expressed by researchers. This finding also poses challenges to biodiversity conservation in the region, as the use of native species may enhance their protection by local population as valuable resources, but further studies are needed, which would give more insight into management practices, in order to state whether these resources are actually over-exploited and to suggest some sound actions for the preservation of medicinal native species.

The Guarani people and *Criollos* may look more similar at first sight, as these groups use considerably more medicinal plant species than Polish migrants do, but when we have a closer look at how these medicinal species are used, and which illnesses are cured with plants, we learn that actually *Criollos* and Polish migrants are more similar to each other, due to the same symptom-specific approach of treating illnesses and probably due to a greater knowledge exchange between the two groups. Therefore the richness and diversity of medicinal plants used in home medicine is built upon intimate and prolonged contact with the environment and through constant trial-and-error effort, but knowledge transfer is facilitated by sharing the ethnomedical corpus of knowledge and procedures of plant inclusion. As previously it was explained, Guarani people are guided by cultural meanings and their cosmovison when incorporating new plant species into their pharmacopeia.

The culturally most important species are different for all study groups. For the Guarani these are almost exclusively native species, while for *Criollos* they are both native and introduced species and for Polish people these are exclusively exotic species. These differences are coupled with a relatively low similarity of medicinal plants between the study groups. This may indicate that Guarani and *Criollos* have their own, culturally sensitive, modes of inclusion of medicinal plants and these two groups have explored, mostly independently, the environment in search of remedies. Polish people have been the most receptive to new plant remedies in the host country, as supported by the fact of the prevalence of native Argentinean species in their pharmacopoeia and the largest similarity in medicinal plant use with *Criollos*, with whom they had the most intense contact since their arrival in Misiones [26]. Therefore, the conclusion here may be that for a migrant group, for whom phytotherapy played the most important part in folk medicine in their country of origin, settling in a highly bio-diverse region with a rich native phytotherapy tradition is a crucial factor in the re-creation and enrichment of their own pharmacopeia. However, the flux of knowledge in this circumstances cannot be equitable.
